# Supplementary material for: Lipoprotein DolP supports proper folding of BamA in the bacterial outer membrane promoting fitness upon envelope stress
Source: eLife. 2021 Apr 13;10:e67817. doi: 10.7554/eLife.67817 (PMC8081527; doi:10.7554/eLife.67817)
Supplement: Supplementary file 1. [file elife-67817-supp1.docx]

**Supplementary file 1**: List of strains used in this study

| **Name** | **Genotype and relevant features** | **Source** |
| --- | --- | --- |
| BW25113 | Δ(araD-araB)567 Δ(rhaD-rhaB)568 ΔlacZ4787(::*rrnB-3*) hsdR514 rph-1 (wild-type reference) | (Grenier et al., 2014) |
| Δ*rseA* | BW25113 rseA::kan | This study |
| Δ*dolP* | BW25113 dolP::kan | This study |
| Δ*bamB* | BW25113 bamB::kan | This study |
| Δ*bamB* Δ*dolP* | BW25113 Δ*bamB* dolP::kan | This study |
| ΔdolP ΔrseA | BW25113 ΔdolP rseA::kan | This study |
| Δskp | BW25113 skp::kan | This study |
| ΔdolP Δskp | BW25113 ΔdolP skp::kan | This study |
| ΔdegP | BW25113 degP::kan | This study |
| ΔdolP ΔdegP | BW25113 ΔdolP degP::kan | This study |
| dolP-gfp | BW25113 dolP-gfp | This study |
| ΔsurA | BW25113 surA::kan | This study |
| ΔompA | BW25113 ompA::kan | This study |
| ΔsurA dolP-gfp | BW25113 surA::kan dolP-gfp | This study |
| ΔbamB dolP-gfp | BW25113 bamB::kan dolP-gfp | This study |
| ΔompA dolP-gfp | BW25113 ompA::kan dolP-gfp | This study |
| ΔompC dolP-gfp | BW25113 ompC::kan dolP-gfp | This study |
| ΔdolP  bamD-mCherry | BW25113 ΔdolP bamD-mCherry | This study |
| bamD-mCherry | BW25113 bamD-mCherry | This study |
| *dolP-gfp*  nlpD-mCherry | BW25113 *dolP-gfp* nlpD-mCherry | This study |
| ΔsurA *dolP-gfp*  nlpD-mCherry | BW25113 *dolP-gfp* nlpD-mCherry surA::kan | This study |
| *dolP-gfp*  zipA-mCherry | BW25113 *dolP-gfp* zipA-mCherry | This study |
| JCM166 | MC4100 *ara^r/-^* Δ(*λatt-lom*)::*bla* P*_BAD_yaeT araC* Δ*yaeT* (BamA depletion strain) | (Wu et al., 2005) |
| LC-E75 | F^–^ λ^–^ *ilvG*^–^ *rfb-50* *rph-1* *186attB*::P*_tet_-dcas9*, λ*attB::mCherry* | (Cui et al., 2018) |
| LC-E75 Δ*dolP* | LC-E75 *dolP::kan* | This study |
