# Supplementary material for: Lipoprotein DolP supports proper folding of BamA in the bacterial outer membrane promoting fitness upon envelope stress
Source: eLife. 2021 Apr 13;10:e67817. doi: 10.7554/eLife.67817 (PMC8081527; doi:10.7554/eLife.67817)
Supplement: Supplementary file 2. [file elife-67817-supp2.docx]

**Supplementary file 2**: List of plasmids used in this study

| **Name** | **Relevant features** | **Source** |
| --- | --- | --- |
| pCtrl | Reference empty vector for ectopic protein expression | This study |
| pBAM | Ectopic expression of all 5 BAM subunits | This study |
| pBAM^His^  (pJH114) | Ectopic expression of all 5 BAM subunits; BamE is C-terminally His-tagged | (Roman-Hernandez et al., 2014) |
| pBAM^ProtA^ | Ectopic expression of all 5 BAM subunits; BamE is C-terminally Protein A-tagged; a TEV site amino acid linker is positioned immediately upstream of the tag | This study |
| pBAM^ΔP1/ProtA^ | Ectopic expression of all 5 BAM subunits; BamA harbours the deletion of POTRA1 and BamE is C-terminally Protein A-tagged; a TEV site amino acid linker is positioned immediately upstream of the tag | This study |
| pBAM^ΔP2/ProtA^ | Ectopic expression of all 5 BAM subunits; BamA harbours the deletion of POTRA2 and BamE is C-terminally Protein A-tagged; a TEV site amino acid linker is positioned immediately upstream of the tag | This study |
| pBamABDE^His^ | Ectopic expression of BamA, BamB, BamD and BamE subunits; BamE is C-terminally His-tagged | This study |
| pBamACDE^His^ | Ectopic expression of BamA, BamC, BamD and BamE subunits; BamE is C-terminally His-tagged | This study |
| pBamBCDE^His^ | Ectopic expression of BamB, BamC, BamD and BamE subunits; BamE is C-terminally His-tagged | This study |
| pBamCDE^His^ | Ectopic expression of BamC, BamD and BamE subunits; BamE is C-terminally His-tagged | This study |
| pBamA | Ectopic expression of wild-type BamA | This study |
| pBamA^His^ | Ectopic expression of C-terminally His-tagged BamA | This study |
| pBamA^ΔP1/His^ | Ectopic expression of a C-terminally His-tagged BamA variant lacking POTRA1 | This study |
| pBamA^ΔP2/His^ | Ectopic expression of a C-terminally His-tagged BamA variant lacking POTRA2 | This study |
| pBamA-DolP^His^ | Ectopic expression of BamA and C-terminally His-tagged DolP. Where indicated, the sequence of the *dolP* open reading frame was mutated to introduce an amber codon in place of the specified position | This study |
| pBamA^ΔP1^-DolP^His^ | Ectopic expression of a BamA variant lacking POTRA1 and C-terminally His-tagged DolP | This study |
| pBamA^ΔP2^-DolP^His^ | Ectopic expression of a BamA variant lacking POTRA2 and C-terminally His-tagged DolP | This study |
| pDolP | Ectopic expression of wild-type DolP | This study |
| pDolP^ProtA^ | Ectopic expression of C-terminally protein A-tagged DolP; a TEV site amino acid linker is positioned immediately upstream of the tag | This study |
| pDolP^His^ | Ectopic expression of C-terminally His-tagged DolP. Where indicated, the sequence of the *dolP* open reading frame was mutated to introduce an amber codon in place of the specified position | This study |
| pDolP^His^-BamA | Ectopic expression of C-terminally His-tagged DolP and BamA. Compared to pBamA-DolP^His^, this plasmid allows a more efficient overproduction of DolP. | This study |
| pOmpA^His^ | Ectopic expression of C-terminally His-tagged OmpA | This study |
| psgRNAcos | Reference empty vector for expression of sgRNAs | (Cui et al., 2018) |
| psgRNA*bamD*  (pCAT187) | Expression of a *bamD*-specific sgRNA | This study |
| psgRNA*bamE*  (pCAT189) | Expression of a *bamE*-specific sgRNA | This study |
| psgRNA*envC*  (pCAT191) | Expression of a *envC*-specific sgRNA | This study |
| psgRNA*ftsX*  (pCAT193) | Expression of a *ftsX*-specific sgRNA | This study |
| pEVOL-pBpF | Expression of tRNA synthetase and tRNA for the *in vivo* incorporation of Bpa at protein positions encoded by an amber codon | (Chin et al., 2002) |
| pYM10 | TEV site and the tandem Protein-A tagging construct | (Knop et al., 1999) |
